# Urine bioassay optimisation to assess the association between antimicrobial exposure, pneumococcal carriage and antimicrobial resistance among hospitalised children in Malawi

**DOI:** 10.1186/s12879-025-11871-w

**Published:** 2025-10-13

**Authors:** Susann Skovbjerg, Comfort Brown, Andrew A. Mataya, Jacquline Msefula, Farouck Bonomali, Faith Banda, Akuzike Kalizang’oma, Robert S. Heyderman, Neil French, Todd D. Swarthout

**Affiliations:** 1 Department of Infectious Diseases, Institute of Biomedicine, University of Gothenburg, Gothenburg, Sweden; 2 Department of Clinical Microbiology, Sahlgrenska University Hospital, Gothenburg, Region Västra Götaland Sweden; 3 Centre for Antibiotic Resistance Research, University of Gothenburg, Gothenburg, Sweden; 4 Malawi-Liverpool-Wellcome Research Programme, Blantyre, Malawi; 5 NIHR Global Health Research Unit on Mucosal Pathogens, Research Department of Infection, Division of Infection and Immunity, University College London, London, UK; 6 Department of Pathology, School of Medicine and Oral Health, Kamuzu University of Health Sciences, Blantyre, Malawi; 7 Centre for Global Vaccine Research, Institute of Infection and Veterinary & Ecological Science, University of Liverpool, Liverpool, UK

**Keywords:** Streptococcus pneumoniae, Acute respiratory infection, Children, Antibiotics

## Abstract

**Background:**

Understanding antibiotic exposure in low-income settings is crucial when designing and evaluating field research on bacterial carriage and antimicrobial resistance. We evaluated the concordance between reported pre-hospitalisation antibiotic use and antimicrobial activity in urine from children presented with acute respiratory infection (ARI) at a Malawian referral hospital. We further investigated the association between antibiotic exposure and antibiotic resistance prevalence among nasopharyngeal pneumococci.

**Methods:**

Between November 2016 and July 2019, 102 children aged 1–4 years with ARI were recruited at Queen Elizabeth Central Hospital in Blantyre, Malawi. Nasopharyngeal swab and urine were collected prior to hospital administered antibiotics. History of antibiotic exposure was obtained by questioning the caregiver and reviewing patient-retained medical records. An optimised urine bioassay was used to detect antimicrobial activity in urine. Pneumococci isolated from nasopharyngeal swabs were tested for antibiotic susceptibility by disc diffusion and E-tests.

**Results:**

Antimicrobial activity from urine specimens were detected in 64% (65/102) of children. Among 29 children with no reported or no recorded pre-admission antibiotic treatment, antimicrobial activity was detected in 11 cases (38%). Among the 73 children with reported antibiotic treatment before admission, 54 (74%) had antimicrobial activity in urine. Pneumococcal carriage prevalence was 63% in children with pre-admission antibiotics based on parent reports and 62% in those without reported pre-treatment. Based on results from the bioassay the pneumococcal prevalence was 57% in children with antimicrobial activity in urine and 73% in those without activity (*p* = 0.11). No difference was found in the pneumococcal antibiotic susceptibility in children with or without urine antimicrobial activity.

**Conclusion:**

This urine bioassay could be a useful and non-invasive tool for objective assessment of pre-clinic antibiotic treatment, as questionnaires can underestimate antibiotic exposure. This could impact surveillance strategies of bacterial carriage prevalences. A short detection period of some antibiotics in urine may limit the use of the bioassay. We found no signs of selection of resistant pneumococcal strains in children with antimicrobial activity in urine.

**Supplementary Information:**

The online version contains supplementary material available at 10.1186/s12879-025-11871-w.

## Introduction

The World Health Organization (WHO) declared antimicrobial resistance (AMR) as one of the top global public health threats with 4.95 million deaths estimated to be associated with bacterial AMR in 2019, most of which occurred in sub-Saharan Africa [1]. Understanding antibiotic exposure is critical in any sick child as it may be used to determine diagnostic yield and the risk of AMR, thus informing an effective treatment strategy. Beyond the relevance for direct patient care, understanding antibiotic exposure and bacterial resistance is a key element of surveillance to inform policy on the broader antimicrobial stewardship activities.

Assessment of pre-admission antibiotic exposure is often based on recall by adult patients or a child’s caregivers [2, 3]. However, such reports of pre-admission antibiotic treatment are often not reliable [4–6]. This can be due to poor recall, denial by interviewee due to self-perceived misuse of medication, an inability to differentiate antibiotics from symptomatic treatment, or the dispensing of antibiotics mixed with other medications in the informal sector [5, 7]. Detection of antimicrobial activity in blood or urine by bioassays, offers an objective assessment of antibiotic exposure [6]. By detecting antimicrobials in urine from patients visiting hospitals in Laos, for example, investigators determined the frequency of antibiotic use to be significantly higher than that reported by patients [4], similar to findings in the Philippines [5].

There are few data on the use of bioassays for detection of antimicrobial activity in urine from children seeking health care in sub-Saharan Africa. In The Gambia, a urine bioassay was used to detect antimicrobial activity in 71% of children presenting to hospital with suspected severe infection [8]. In Ghana, patients of all ages visiting health facilities for bacteriological laboratory test were less likely to have a positive bacterial culture if antimicrobial activity was detected in their urine [9]. In Northern Uganda, 19% of adults who visited outpatient departments and reported not taking any antibiotics at home had antibacterial activity in urine [10]. In South Africa, children recruited to the Pneumonia Etiology Research for Child Health (PERCH) study were sampled for antimicrobial activity in urine and serum [11]. The broad conclusion from PERCH was that parental reporting was not sufficiently reliable and, subsequently, the PERCH analysis largely relied on antibiotic treatment data from referral facilities.

Across much of sub-Saharan Africa, pneumococcal disease (including pneumonia, bacteraemia and meningitis) contributes significantly to health facility visits and admissions, leading to antibiotic exposure in children [12]. Moreover, resistant *Streptococcus pneumoniae* is a leading pathogen attributable to AMR associated death in sub-Saharan Africa [13]. Malawi introduced the 13-valent pneumococcal conjugate vaccine (PCV13) into the routine infant immunization program in November 2011. Despite PCVs contributon to reduced pneumococcal disease incidence and associated hospitalisation, as shown in Malawi and Kenya [14, 15], bacteria (including *Streptococcus pneumoniae*) still predominate in the very severe cases of childhood pneumonia [16].

The reported prevalence of pneumococcal carriage among children hospitalised with respiratory illness is lower than among otherwise healthy children, including in Mozambique (45% versus 84%, respectively) [2] and in the Democratic Republic of (DR) Congo [3, 17]. This is likely due to antibiotic exposure prior to admission [18]. For example, 87% of parents of hospitalised Congolese children reported antibiotic use shortly before their child was hospitalised [3], compared with 20% of parents of non-hospitalised children (i.e. attending a health centre for immunisation or growth monitoring) [19]. Similarly in Mozambique, 83% of parents of children hospitalised with pneumonia reported recent antibiotic use, compared to 27% of parents of children without pneumonia [2]. While adequate antibiotic treatment is unquestionably essential for treating children with bacterial pneumonia [20], there is growing evidence of antibiotic over-prescription [21]. Moreover, though regulated as prescription-only in many low- and middle-income countries, including Malawi, antibiotics are commonly available without prescriptions from private pharmacies and drug stores [22]. Consequently, children seeking care at hospitals are often treated with antibiotics prior to admission, either by prescription from a primary health care centre or administration by caregivers.

In this study, using sampling from an established study [23], we aimed to [1] assess the concordance between the child’s pre-admission antibiotic treatment (as reported by caregivers, with or without reference to patient-retained health documents) and the detection of antimicrobial activity in the child’s urine; [2] report on the optimisation of an easily implemented urine bioassay and its robustness in a low-income setting; and [3] assess if antibiotic exposure is associated with differences in pneumococcal carriage prevalence and antibiotic susceptibility in hospital-admitted children.

## Materials and methods

### Study setting and patients

Queen Elizabeth Central Hospital (QECH), located in the urban city of Blantyre, is a tertiary referral hospital for the district hospitals of the Southern Region of Malawi. It secondarily functions as a district hospital for Blantyre, providing free medical care to the district’s 1.3 million urban, peri-urban, and rural residents.

This study was based on data collected during a larger community-based pneumococcal carriage surveillance project [23]. Eligible study participants included children aged 1–4 years admitted to QECH due to acute respiratory infection (ARI) between November 2016 and July 2019, and who could provide a urine sample. After the caregiver provided written informed consent, the caregiver collected a urine sample from the child. This was performed at the paediatric inpatient ward and did not delay any treatment nor investigation of severely ill children. Those children receiving antibiotic treatment at the hospital before urine sample collection was excluded from the final analysis. Nasopharyngeal (NP) swab samples were collected from all recruited children, as described below. The caregiver was questioned about the child’s pre-hospitalisation antibiotic exposure, including any treatment at a primary health care centre or acquired from any other source during the 7 days before hospital admission (Supplementary file 1). If the child’s health passport (patient-retained medical record) was available, it was reviewed for further information about antibiotic prescriptions prior to hospitalisation. The analysis did not differentiate whether antibiotic data were collected from the caregiver alone (recall), from the health passport alone, or from both. Inpatient medical files were used to assess whether antibiotics were given to the child post-admission but before urine collection. After 12 October 2018, the questionnaire was amended to collect information about [1] the date and time of hospital-administrated antibiotics and [2] the date and time of urine collection as noted in the study Standard Operating Procedure (Supplementary file 2). Hospital forms were reviewed to capture the child’s diagnosis at admission.

### Urine sampling

Urine was either collected into a urine bag or passed directly into a sterile 25-mL container. The sample was kept in a cooler box (2–8 °C) until delivery to the Malawi Liverpool Wellcome Programme (MLW) laboratory, co-located with QECH. After the sample was vortexed for 10 s, two aliquots of urine (each 1.5–1.8 mL) were archived on the same day at − 80 °C.

### Nasopharyngeal swab collection

An NP swab sample was collected from each participating child. The method for collecting study swabs is described elsewhere [23]. In brief, each NP swab sample was collected using a nylon flocked swab (FLOQSwabs; Copan Diagnostics, Murrieta, CA, USA), immediately placed in 1.5 mL skim milk-tryptone-glucose-glycerol (STGG) medium and kept in a cooler box (2–8 °C) until delivery to the MLW laboratory. Samples were processed at the laboratory according to WHO recommendations [24]. Samples were frozen at − 80 °C within 8 h of collection.

### NP sample culture

After a frozen NP-STGG sample was allowed to thaw completely on ice, it was vortexed for 10 s, and then a sterile inoculation loop was used to streak 30 µL of sample in a 3-quadrant fashion onto sheep blood agar (7%) supplemented with 5 µg/mL gentamicin (SBG-agar, prepared in-house). An optochin disc (Oxoid, Thermo Scientific, Basingstoke, Hampshire, UK) was applied and the plate was incubated for 18–24 h at 35–37 °C with ~ 5% CO_2_. Colonies with typical pneumococcal morphology and with optochin inhibition zones ≥ 14 mm were identified as *S. pneumoniae*. Suspected colonies that were optochin resistant (optochin inhibition zone < 14 mm) or indeterminant were further evaluated with the bile solubility test. If no pneumococci were identified, the agar plate was re-incubated for 18–24 h (35–37 °C, ~ 5% CO2) before it was documented as negative if no growth was seen. When *S. pneumoniae* was confirmed, a single colony was picked and cultured on a new SBG-agar plate with an applied optochin disc and incubated as described above. Serotyping of confirmed pneumococcal growth was performed using the 13-valent latex test (Statens Serum Institut, Copenhagen, Denmark) according to the manufacturer’s instructions. The remaining bacteria were harvested and archived in Microbank (Pro-Lab Diagnostics, Birkenhead, UK) cryovials at − 80 °C.

### Antimicrobial susceptibility testing of isolated pneumococci

Pneumococcal isolates were tested for antimicrobial susceptibility by use of the disk diffusion method (Oxoid, Thermo Scientific) and E-tests (BioMérieux, Marcy l’Etoile, France). Archived *S. pneumoniae* isolates were cultured on sheep blood agar and incubated overnight, as described above. A sterile cotton swab was used to suspend a sweep of colonies in sterile saline to the density of a McFarland 0.5 standard. A sterile cotton swab was then used to spread the bacteria evenly and entirely over the surfaces of two Mueller Hinton agar plates (supplied with 5% sheep blood and 20 mg/L β-nicotinamide adenine dinucleotide, Applichem, Darmstadt, Germany). Sterile forceps were used to apply discs respectively impregnated with the following antibiotics onto the first plate: oxacillin (1 µg), clindamycin (2 µg), erythromycin (15 µg), tetracycline (30 µg), and trimethoprim-sulfamethoxazole (1.25/23.75 µg). The minimal inhibitory concentration (MIC) of benzylpenicillin was determined by applying an E-test strip (0.016–256 µg/mL) onto a second plate. Both plates were then incubated at 35–37 °C with ~ 5% CO_2_ for 16–20 h. Zones of inhibition were measured and interpreted using clinical breakpoints published by the European Committee on Antimicrobial Susceptibility Testing (EUCAST) in 2024 (https://www.eucast.org/fileadmin/src/media/PDFs/EUCAST_files/Breakpoint_tables/v_14.0_Breakpoint_Tables.pdf.).

### Urine bioassay for detecting antimicrobial activity

A bioassay for detecting antimicrobial activity in urine was performed as previously described with some modifications [4, 7, 25, 26]. Briefly, a strain each of *Geobacillus stearothermophilus* (ATCC 7953), *Streptococcus pyogenes* (ATCC 19615), and *Escherichia coli* (ATCC 25922) was seeded on respective Mueller Hinton agar plates or, in the case of *S. pyogenes*, Mueller Hinton agar plates supplemented with 5% sheep blood, all prepared in-house. Up to six autoclaved 6 mm discs (GE Healthcare Life Sciences, Buckinghamshire, UK) were applied onto each agar plate with sterile forceps. After one aliquot of urine was allowed to fully thaw, it was vortexed for 10–15 s, and then 5 µL of urine was applied in duplicates onto the discs of each plate. The volume of 5 µL was found to be optimal after an earlier evaluation of 3, 5, 10, and 20 µL urine volume. The agar plates were incubated for 18–24 h under the following conditions: *E. coli* in a 35–37 °C aerobic milieu, *S. pyogenes* in 35–37 °C with ~ 5% CO_2_, and *G. stearothermophilus* (sealed in a plastic bag) in an aerobic environment at 55 °C. Visual evidence of any growth inhibition around a disc on any plate was taken as evidence of antimicrobial activity in the urine. The bioassay was first performed on urine from 15 children with confirmed antibiotic exposure after admission and prior to urine collection, all showing antimicrobial activity with the bioassay. For each run, a positive control (urine from one of these 15 children) and a negative control (urine from a healthy child with no history of antibiotic exposure within the 8 weeks prior to urine collection and that sample being negative for any antimicrobial activity) were analysed. Weekly quality control tests were performed using the following commercial antibiotic discs: amoxicillin-clavulanic acid (20/10 µg) (Mast Group, Merseyside, UK), ampicillin (10 µg), ceftriaxone (30 µg), chloramphenicol (30 µg), ciprofloxacin (5 µg), erythromycin (15 µg), gentamicin (10 µg), nalidixic acid (30 µg), penicillin G (10 units), vancomycin (30 µg) (all from Oxoid, Thermo Scientific). For *E. coli*, the targets and ranges of the zone diameters for each antibiotic were defined by EUCAST (Version 10.0, 2020) (https://www.eucast.org/fileadmin/src/media/PDFs/EUCAST_files/QC/v_10.0_EUCAST_QC_tables_routine_and_extended_QC.pdf.). There were no targets or zone ranges available for *S. pyogenes* or *G. stearothermophilus*. However, the median variation in zone diameters was found to be 10% for these strains and did not vary more than 13% for any of the antibiotics assessed.

### Assessment of antibiotic stability in urine

The stability of antibiotics detected in the urine was evaluated by subjecting 300-µL urine aliquots from 10 children with known antimicrobial exposure to repeated freeze-thaw cycles. This included 1 month of repeated cycling daily during weekdays (Monday through Friday) from − 80 °C to 1–2 h at ambient room temperature (~ 22 °C, including full thaw) and back to − 80 °C. The samples were analysed by the bioassay once weekly or once every 2 weeks. Another 300-µL aliquot from each of the same samples was continuously kept at the same ambient room temperature (~ 22 °C) and analysed by the bioassay after 5, 13, 20, and 34 days.

### Statistics

#### Sample size calculation

Using the first 33 urine samples with available NP pneumococcal culture results, the pneumococcal carriage prevalence was 55% in children with urinary antimicrobial activity and 70% in children without urinary antimicrobial activity. Using this interim analysis, we calculated that to be able to detect a 15% difference in carriage prevalence (due to antibiotic exposure) with 80% power, a total sample size of 163 in each group was required, using a one-sided test with an alpha value of 0.5 (326 samples in total).

Categorical variables are presented as frequencies and percentages, while age data are presented as medians with interquartile ranges (IQRs). The chi-square test (*Χ* ^2^) was used to compare proportions of children with pneumococcal growth in relation to antibiotic treatment or antimicrobial activity in urine. Statistical calculations were performed using GraphPad Prism 10.2.3 (GraphPad Software, Boston, MA, USA).

## Results

### Study participants

**Fig. 1:**
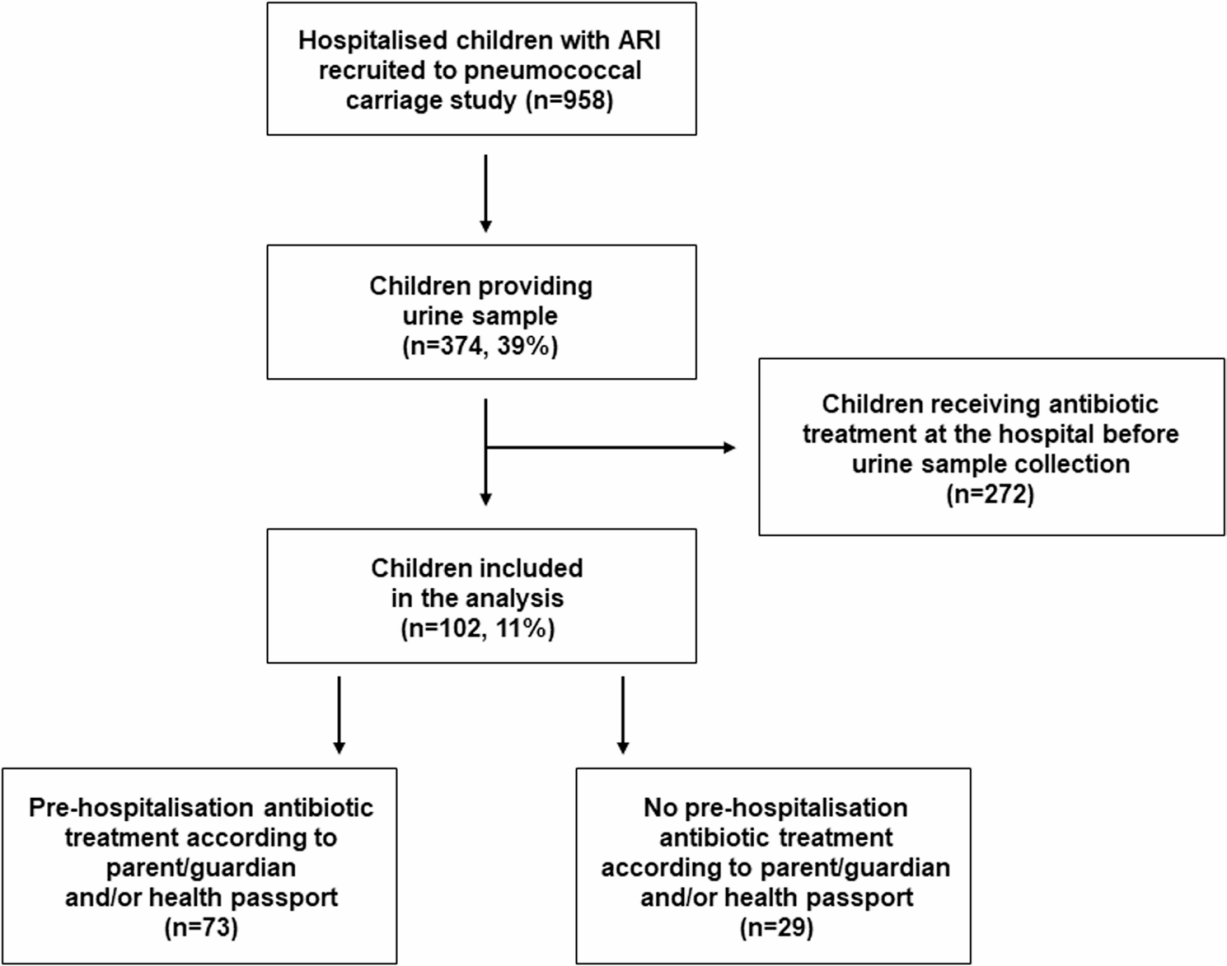
Flow chart of children included in the study. Participants included children 1–4 years old and admitted to Queen Elizabeth Central Hospital in Blantyre, Malawi, due to acute respiratory infection between November 2016 and July 2019. Children were recruited as part of a larger community-based pneumococcal carriage surveillance project [23]

From November 2016 through July 2019, 958 patients were recruited to the established study, among whom 39% (374/958) provided urine samples. Of these, 73% (272/374) received antibiotics at the hospital before a urine sample was collected. Thus, 11% (102/958) of recruited children were included in the final analysis (Fig. 1). The median age was 3.0 (IQR: 1.9–4.1) years, and 62% were male (Table 1). Based on data from either the parents/guardians or available health passports, 72% (73/102) had been treated with antibiotics during the week prior to hospital admission. In most cases (66/73, 90%), treatment occurred at primary health care centres (Table 1). Despite the high frequency of pre-admission antibiotics, 67% (68/102) of children presented with clinical symptoms suggesting non-infectious (including asthma) or viral (including bronchiolitis) diagnoses. Pneumonia or severe pneumonia was suspected in 17% (17/102) of the patients at admission (Table 1). The proportion of children receiving pre-admission antibiotics did not differ between the 102 children in the final analysis (72%) and the 272 excluded children (76%) (*p* = 0.33) (Table 1). Benzylpenicillin was the most frequently used pre-admission antibiotic (63/73, 86%), sometimes combined with other antibiotics, including gentamicin (18/73, 25%) (Table 2). Eighteen children (18/73, 25%) received co-trimoxazole, and 16 children (22%) received amoxicillin (Table 2).

**Table 1:** Participant characteristics

	No antibiotics received at QECH prior to urine collection (*n* = 102)^a^	Antibiotics received at QECH prior to urine collection (*n* = 272)
Age years, median (IQR)	3.0 (1.9–4.1)	2.7 (1.8–3.7)
Sex, male n (%)	63 (62)	164 (60)
Referred from HC to QECH	84 (82)	217 (80)
Antibiotics received pre-admission^b^	73 (72)	208 (76)
Received at HC^b, c^	66 (65)	171 (63)
Source other than HC^b, c^	30 (29)	98 (36)
Clinical diagnosis at admission^d^		
Viral respiratory infection^e^	33 (32)	--^f^
Pneumonia/Severe pneumonia	17 (17)	--
Asthma	28 (27)	--
Bronchiolitis Other^g^ No diagnosis	20 (20) 5 (4.9) 15 (15)	--

*IQR* Interquartile range, *HC *health centre, *QECH *Queen Elizabeth Central Hospital

^a^Included in urine bioassay analysis

^b^ According to the parent/guardian and/or the health passport

^c^ Participant could have received antibiotics from multiple sources, including from health centre or from ‘other source’

^d^ More than one diagnosis possible for each child

^e^ Includes “Viral induced wheeze” and “Viral respiratory infection”

^f^ Data not available

^g^ Including malaria/severe malaria, tracheal tumour, coryza, extrapulmonary tuberculosis, immune suppression, upper respiratory tract infection, febrile convulsions

**Table 2:** Urinary bioassay results among children with no post-admission antibiotic exposure prior to urine collection (*n* = 102)

Antibiotic history, as reported by parent/guardian or health passport	Antimicrobial activity (*n* = 65) *n* (%)	No antimicrobial activity (*n* = 37) *n* (%)
Pre-admission antibiotics (*n* = 73)	54 (74)	19 (26)
Received from HC only (*n* = 43)	31 (72)	12 (28)
Benzylpenicillin	31	10
Gentamicin	7	6
Amoxicillin	1	1
Co-trimoxazole	0	1
Received from HC and other source (*n* = 23)	17 (74)	6 (26)
Benzylpenicillin	16	6
Gentamicin	3	2
Amoxicillin	9	2
Co-trimoxazole	10	4
Chloramphenicol	0	1
Received from other source only (*n* = 7)	6 (86)	1 (14)
Amoxicillin	3	0
Co-trimoxazole	2	1
Erythromycin	1	0
No pre-admission antibiotics (*n* = 29)	11 (38)	18 (62)

*HC* Health centre

### Antimicrobial activity in urine

Antimicrobial activity was detected in the urine of 64% (65/102) of children. Among the 73 children with reported antibiotic treatment within 1 week before admission, 54 (74%) had antimicrobial activity in urine; the other 19 urine samples (26%) showed no antimicrobial activity (Table 2). In contrast, among the urine samples from the 29 children with no reported history of pre-admission antibiotics, 38% (11/29) had antimicrobial activity. The bioassay was designed to detect antimicrobial activity generated by all but one of the antibiotics reportedly taken by the enrolled children; the exception was chloramphenicol, which had been given to one child.

In all but one child (64/65, 98%) with antimicrobial activity detected in the urine, inhibition zones emerged around discs on the agar plates seeded with *G. stearothermophilus*, whereas inhibition zones were visible on *S. pyogenes* and *E. coli* plates in 45% and 31% of the positive cases, respectively. The median zone diameters were 23 mm (IQR: 17–32 mm) for *G. stearothermophilus*, 14 mm (IQR: 10–24 mm) for *S. pyogenes*, and 11 mm (IQR: 9–13 mm) for *E. coli.* Similar results were obtained with all duplicate samples tested on each of the strains; the zone diameters for *G. stearothermophilus* did not differ by more than 4 mm, and the maximum variations between the duplicates were 1 and 2 mm for *S. pyogenes* and *E. coli*, respectively.

### Stability of antibiotics in the urine

**Fig. 2:**
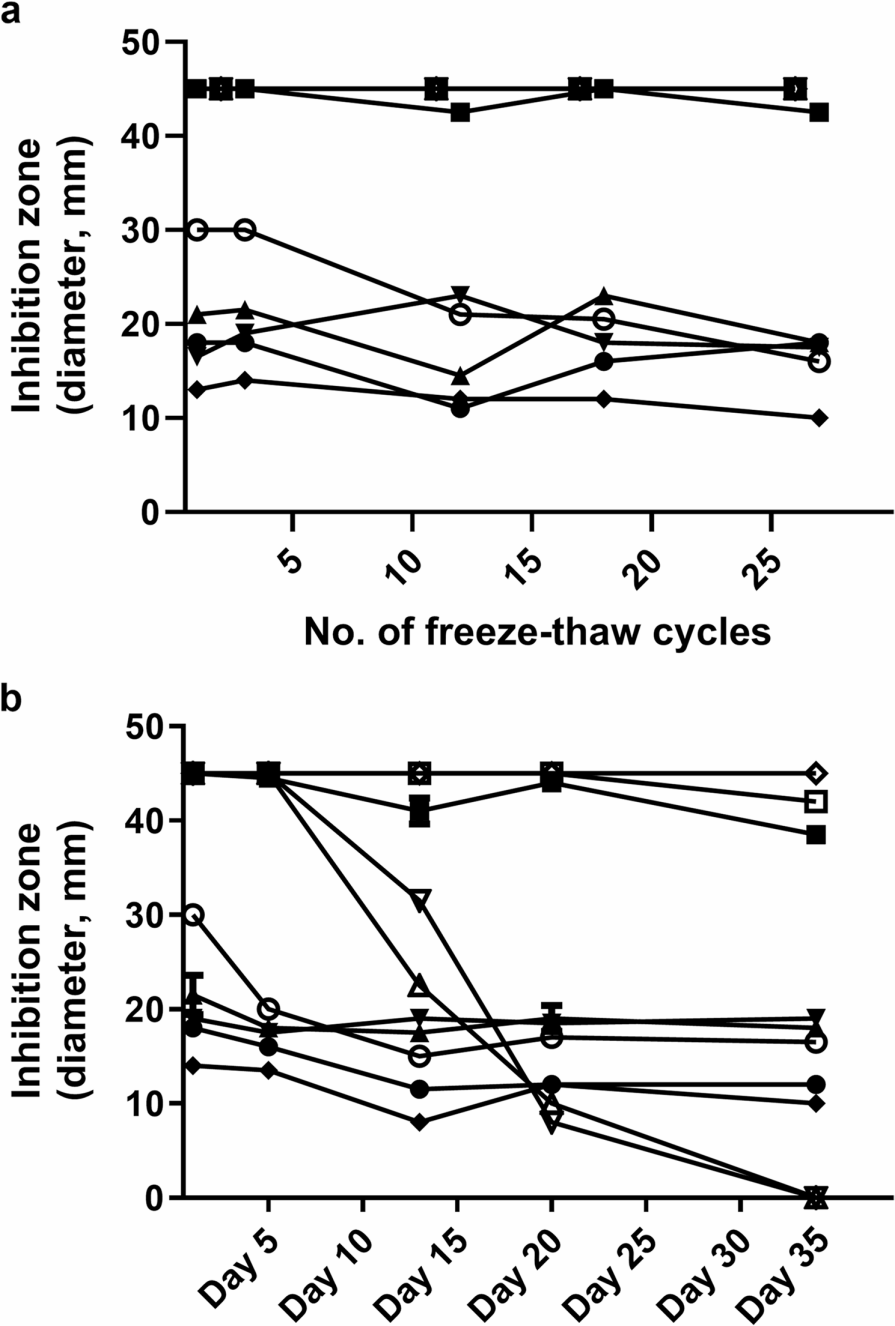
Urine antibiotic stability test results. **a** Urine bioassay inhibition zone diameters (mm), using the *G. stearothermophilus* (ATCC 7953) strain, of 10 urine samples challenged to multiple freeze-thaw cycles. Each symbol represents one urine sample obtained from a child. Note: Four samples have overlapping lines, given they have all maintained maximum values throughout the test. **b** Urine samples from 10 children were stored at room temperature and analysed weekly for antimicrobial activity by the urine assay for just over a month´s period. The inhibition zone diameters (mm) of only the *G. stearothermophilus* (ATCC 7953) strain are shown. One sample maintained maximum values during the observation period

To assess the stability of the antibiotics and the robustness of the bioassay, 300-µL urine samples from 10 children with known antimicrobial activity were subjected to 27 freeze-thaw cycles over 1 month. Antimicrobial activity was consistently detected from all 10 samples after the freeze-thaw events (Fig. 2a). Among the separate 300-µL aliquots from the same 10 samples kept at room temperature and analysed each week just over one month, all samples were positive until day 20, and eight samples continued to show antimicrobial activity for 34 days (Fig. 2b).

### Association between pneumococcal carriage and antibiotic exposure

The pneumococcal carriage prevalence was similar between children who were reportedly treated with antibiotics pre-admission and those who were not (63% versus 62%, respectively) (Table 3). However, there was a trend towards lower pneumococcal carriage prevalence among children with evidence of urinary antimicrobial activity compared to those with no antimicrobial activity (57% versus 73%, *Χ* ^2^ *p* = 0.11). Thus, the carriage prevalences tended to differ slightly whether pre-hospitalisation antibiotic exposure was assessed by reports or by detection in urine. Among children who received antibiotics after admission and before NP sample collection, the pneumococcal carriage prevalence was significantly lower (78/270, 29%; data missing for two patients) than among children who did not receive antibiotics in the hospital before NP sample collection (64/102, 63%, *p* < 0.0001). The prevalence of PCV13 serotypes did not differ between children with or without urine antimicrobial activity, with non-vaccine serotypes predominating in both groups (Table 3).

**Table 3:** Association between pneumococcal carriage and pre-hospitalisation antibiotic treatment

	Urinary antimicrobial activity		*p*-value, Χ ^2^	Reported history of antibiotics prior to admission, *n* (%)		*p*-value, Χ ^2^
	Detected (*n* = 65) *n* (%)	Not detected (*n* = 37) *n* (%)		Yes (*n* = 73) *n* (%)	No (*n* = 29) *n* (%)	
Pneumococcal growth	37 (57)	27 (73)	0.11	46 (63)	18 (62)	0.93
VT	6 (16)	4 (15)	0.88	6 (13)	4 (22)	0.36
NVT	31 (84)	23 (85)	0.88	40 (87)	14 (78)	0.36
No pneumococcal growth	28 (43)	10 (27)	0.11	27 (37)	11 (38)	0.93

*NVT* Non PCV13 serotypes, *VT *PCV13 serotypes, *Χ*^2^ chi-squared test

### Antibiotic susceptibility of the colonising pneumococci

**Fig. 3:**
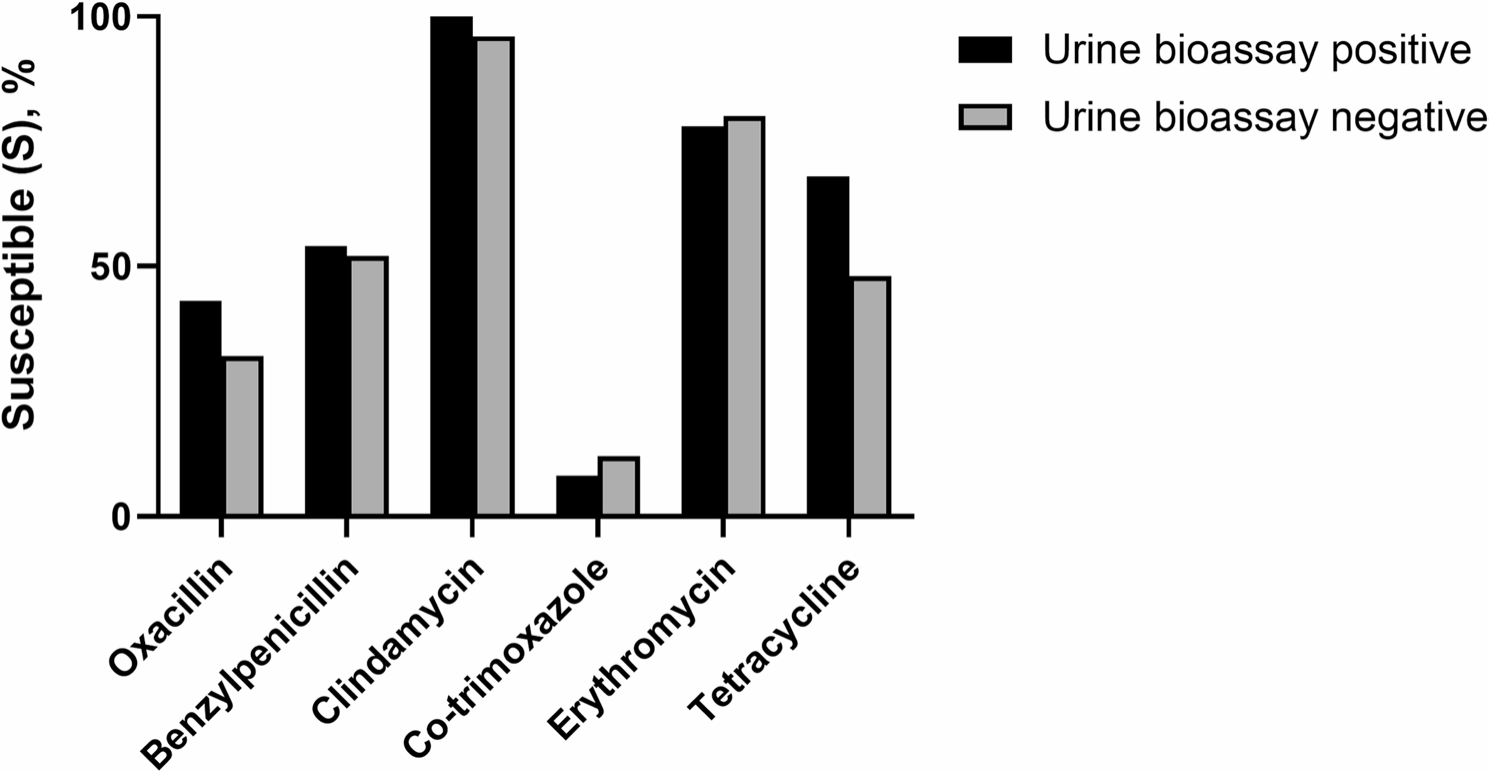
Antibiotic susceptibility. Sixty-two (62) pneumococcal isolates were cultured from children with (*n* = 37) or without (*n* = 25) antimicrobial activity in the urine. Susceptibility to oxacillin, clindamycin, co-trimoxazole, erythromycin, and tetracycline was tested by disc diffusion test. Benzylpenicillin susceptibility was tested by minimum inhibitory concentration (MIC) determination by E-test.

Antibiotic susceptibility was determined among pneumococci isolated from children whose urine was analysed for antimicrobial activity (*n* = 62). There were no significant differences between children with positive versus negative urinary antimicrobial activity in terms of pneumococcal susceptibility to any of the antibiotics evaluated (Fig. 3; Table 4). For all isolates, the median benzylpenicillin MIC was 0.064 mg/L (range: 0.008–1.008); thus, no child carried pneumococci resistant to benzylpenicillin (MIC value threshold = 4 mg/L) for treatment of infections other than meningitis. In 38/62 isolates (61%), the oxacillin zone was < 20 mm (i.e., resistant to phenoxymethylpenicillin and benzylpenicillin when used for meningitis treatment) (Table 4). While almost all isolates (57/62, 92%) were susceptible to ampicillin (oxacillin zone ≥ 9 mm) and clindamycin (98%), the resistance rate against co-trimoxazole was high (90%) (Table 4).

**Table 4:** Antibiotic susceptibility of colonising pneumococci among hospitalised children with or without antimicrobial activity in urine

	Urine bioassay positive (*n* = 37) *n* (%)			Urine bioassay negative (*n* = 25), *n* (%)			Total (*n* = 62), *n* (%)		
	S	I	R	S	I	R	S	I	R
Benzylpenicillin	20 (54)	17 (46)	0	13 (52)	12 (48)	0	33 (53)	29 (47)	0
Oxacillin	16 (43)	-	-	8 (32)	-	-	24 (39)	-	-
Clindamycin	37 (100)	-	0	24 (96)	-	1 (4)	61 (98)	-	1 (2)
Erythromycin	29 (78)	-	8 (22)	20 (80)	-	5 (20)	49 (79)	-	13 (21)
Co-trimoxazole	3 (8)	-	34 (92)	3 (12)	-	22 (88)	6 (10)	-	56 (90)
Tetracycline	25 (68)	-	12 (32)	12 (48)	-	13 (52)	37 (60)	-	25 (40)

The susceptibility of the pneucmococcal isolates to each antibiotic was determined by disc diffusion test, or, to benzylpenicillin, by E-test, using clinical breakpoints published by the European Committee on Antimicrobial Susceptibility Testing (EUCAST) in 2024

*I* Susceptible, increased exposure; *S *Susceptible, standard dosing regimen, *R *Resistant; - = not applicable

## Discussion

Investigating children admitted with ARI to a large referral hospital in Blantyre, Malawi, we determined a 29% discordance between participant-reported antibiotic exposure and exposure detected by a urine bioassay. We also determined a 16% lower pneumococcal carriage prevalence associated with antibiotic exposure confirmed by the same urine bioassay. To arrive at these endpoints, we optimised previously published assay protocols for detecting antimicrobial activity in urine [4, 6, 7, 25, 26]. These findings underline important challenges that need to be considered when designing and evaluating field research on bacterial carriage and AMR.

In our study, antimicrobial activity was detected in about one-third of patients who reported no history of recent antibiotic treatment, lower than the 76% discordance observed in a similar child cohort in the Philippines [5]. In Argentina, concordance in a child’s recent antibiotic treatment was relatively reliable only when the parent’s answer was affirmative [27]. Discordances may be due to poor recall, denial, or parent´s lack of knowledge, i.e. inability to differentiate whether a drug is an antibiotic or a symptomatic drug [5, 7]. Thus, relying only on the caregiver when assessing pre-hospitalisation antibiotic use can lead to significant underestimations, but may also lead to overestimation.

No antimicrobial activity was detected in 26% (19/73) of patients who reported recently receiving pre-hospitalisation antibiotics. The urine bioassay detects beta-lactam antibiotics and erythromycin for about 12–24 h after consumption, with clindamycin, tetracycline, co-trimoxazole and ciprofloxacin detection possible for ≥48 h after a single oral dose [25]. Thus, among urine samples with no antibiotic activity from children who reportedly received an antibiotic pre-hospital, the antibiotic could have been given to the child during the week preceding admission but at a time that exceeded the detection period of 12–24 h.

Eighty-nine per cent (89%) of eligible hospitalised children with ARI were excluded from the main analysis, either because no urine sample was taken or because antibiotics were given at the hospital before the urine sample was taken. Such restrictions can limit the power and thus the interpretation of the results. Though antimicrobial activity can also be detected from blood [6] and would likely facilitate rapid recruitment, urine is a non-invasive specimen. Previous studies also report higher sensitivity with urine bioassays compared with serum-based assays [6, 11], in part because urine concentrations can be 100 to 1000 times greater than concomitant serum antibiotic concentrations [28].

There are concerns about loss of antimicrobial activity in urine if samples are exposed to multiple freeze-thaw cycles [7, 26]. In this study, antimicrobial activity could still be detected in urine after more than 25 freeze-thaw cycles. Storage at room temperature also did not have a detrimental effect on sample viability; after 3 weeks, antimicrobial activity was still detected in all examined samples. This illustrates the robustness of the method, as well as the slow degradation of the antibiotic molecules [29], which is important information for field research in low-income settings, where maintaining a robust cold chain can be a challenge.

In 98% of positive samples, antimicrobial activity was detected using the *G*. *stearothermophilus* strain, similar to previous reports [7, 26]. Thus, the use of only this strain might have been sufficient for this cohort, a factor to consider in resource-limited settings. This strain has the highest sensitivity but also the lowest specificity of the three reference strains [25]. One disadvantage of using *G*. *stearothermophilus* is the need for a 55 °C incubator, a temperature not routinely used in microbiology laboratories. We encountered no cases where antimicrobial activity was detected using *S. pyogenes* alone. There was only one case in which inhibition zones were visible on *E. coli* plates but not on plates seeded with the other two strains. A similar pattern was observed in the Philippines [26], but in other settings a considerable number of patients with antibiotic activity in their urine would have been missed if *G*. *stearothermophilus* alone had been used [4, 30].

Previous work has explored the association between pneumococcal carriage detection and antibiotic treatment. Amoxicillin therapy reportedly halved the proportion of Spanish children colonised with pneumococci, though colonisation prevalence returned to baseline 1 month after treatment [31]. Similarly, antibiotic use within 2 weeks preceding sample collection was negatively associated with pneumococcal carriage in Kenyan children [18]. The PERCH Study determined that antibiotic exposure was associated with significantly reduced pneumococcal carriage detection among both hospitalised children with severe pneumonia and community controls [11].

The carriage prevalence in this study varied depending on whether pre-hospitalisation antibiotic exposure was assessed by patient history or by the bioassay. There was a tendency of lower carriage prevalence among children in whom urinary antimicrobial activity was detected than among those with no detected antimicrobial activity. By comparison, the carriage prevalence was similar between patients with or without pre-hospitalisation antibiotics when antibiotic exposure was assessed by patient information and health passports. This illustrates the need for reliable objective tools when evaluating endpoints that may be influenced by previous antibiotic use.

Children receiving antibiotics in hospital and before NP sample collection (thus excluded from the main analysis) had a lower pneumococcal carriage prevalence (29%) than children not treated with antibiotics before NP sampling but with detected antimicrobial activity in urine indicating recent antibiotic exposure (57%). Most of the hospital-treated children had received benzylpenicillin, sometimes together with gentamicin, suggesting that the pneumococci that had been carried by these children, to a large extent, were susceptible to beta-lactam antibiotics.

In high-income countries, correlations between outpatient antibiotic use and prevalence of penicillin non-susceptible *S. pneumoniae*, as well as between macrolide use and prevalence of macrolide-resistant *S. pneumoniae*, have been observed [32]. Similar findings of correlations of beta-lactam and macrolide use with penicillin-resistant pneumococci, and use of erythromycin with erythromycin-resistant *S. pneumoniae*, were reported from Thailand [33]. In Vietnam, significantly higher frequencies of NP *Haemophilus influenzae*, *S. pneumoniae*, and *Moraxella catarrhalis* resistant to ampicillin and/or penicillin were found in children recently treated with beta-lactam antibiotics [34]. Our study did not find a difference in antibiotic susceptibility between pneumococci isolated from children with versus without antimicrobial activity in the urine. Hence, differences in isolation rates were likely not due to selection of resistant strains.

A limited number of pneumococcal isolates (*n* = 62) were assessed for antibiotic susceptibility. Most pneumococci were susceptible to amoxicillin and ampicillin, similar to findings from Northern Tanzania [35], though unlike pneumococci carried by children in Eastern DR Congo [19]. The WHO recommends amoxicillin as first-line pneumonia treatment for children, while ampicillin plus gentamicin is the first-line treatment for severe pneumonia [36]. Not surprisingly, given the use of co-trimoxazole in Malawi’s national HIV strategy, a high level of resistance against co-trimoxazole was found.

Eligibility criteria included not receiving antibiotics at the hospital before urine collection, likely excluding children with severe bacterial infection. The proportion of reported pre-hospitalisation antibiotic treatment was high and did not differ between included and excluded children. Though this might have been due to non-prescribed antibiotic treatment or consultations at non-professional health facilities, it may also illustrate the challenges of clinical counselling at the primary health centre level, often with limited diagnostic capacity. Research among Malawian outpatients demonstrated a concerning level of under-treatment with antibiotics for children with clinical pneumonia but also considerable over-treatment of children with no need for antibiotics [37]. Studies in northern Tanzania have similarly indicated high levels of antibiotic over-prescription, both at hospitals and primary health centres, among children with signs of a common cold [35, 38]. Most cases of febrile illness among sub-Saharan children are considered to have viral etiology [39–41]. Interviews with prescribing Tanzanian health care workers highlighted the need for updated clinical guidelines, including support and guidance in symptomatic treatment of cases where severe infection has been ruled out [42]. Data from Tanzania further suggest that socioeconomic factors and gender inequality affect health care–seeking behaviours in ways that might facilitate irrational antibiotic use in children from low-income families [43]. There is also evidence for the need to further strengthen knowledge in antibiotic stewardship among health care students, including medical students in settings such as Malawi [44].

There were some limitations to the study. The number of included children did not reach the calculated sample size needed to detect a significant difference in pneumococcal carriage prevalence between children with or without antimicrobial activity in urine. One reason for this was the large number of children receiving antibiotics at hospital before inclusion and urine sampling. We did not control for the source of information on pre-hospitalisation antibiotic treatment, i.e., whether the patient history was confirmed by the health passport. No data were available on the dosage or time of the child´s most recent ingestion of antibiotics before the urine sampling. The time of urine collection has previously been described as the most significant limitation of the assay [6, 25].

## Conclusion

In conclusion, the urine bioassay could be a useful and non-invasive tool for objective assessment of pre-clinic antibiotic treatment, as questionnaires might underestimate antibiotic exposure. This should be considered when designing and evaluating surveillances of bacterial carriage and AMR. However, the use of the bioassay may be limited by a short detection period for some antibiotics in urine.

## Supplementary Information

Supplementary Material 1.

Supplementary Material 2.

## Data Availability

The datasets used and analysed during the current study are available from the corresponding author on reasonable request.

## Abbreviations

AMR: Antimicrobial resistance
ARI: Acute respiratory infection
DR Congo: Democratic Republic of Congo
*E. coli*: *Escherichia coli*
EUCAST: European Committee on Antimicrobial Susceptibility Testing
*G. **stearothermophilus*: *Geobacillus* *stearothermophilus*
IQR: Interquartile range
MIC: Minimal inhibitory concentration
MLW: Malawi Liverpool Wellcome Programme
NP: Nasopharyngeal
PCV: Pneumococcal conjugate vaccine
PCV13: 13-valent pneumococcal conjugate vaccine
PERCH study: Pneumonia Etiology Research for Child Health study
QECH: Queen Elizabeth Central Hospital
*S. pneumoniae*: *Streptococcus pneumoniae*
*S. pyogenes*: *Streptococcus* *pyogenes*
STGG: Skim milk-tryptone-glucose-glycerol
WHO: World Health Organization
*X*^2^: Chi-square test
