# Supplementary material for: Urine bioassay optimisation to assess the association between antimicrobial exposure, pneumococcal carriage and antimicrobial resistance among hospitalised children in Malawi
Source: BMC Infect Dis. 2025 Oct 13;25:1291. doi: 10.1186/s12879-025-11871-w (PMC12516879; doi:10.1186/s12879-025-11871-w)
Supplement: Supplementary file 1 — Supplementary Material 1. [file 12879_2025_11871_MOESM1_ESM.pdf]

Label (PID)		Label (SPINE)

PVCPA CRF
PNEUMO-SURVEILLANCE, QECH
Recruitment Post-Admission (Form 1 of 4)

Screening

#	Question	Response	Variable
1	Today's date (dd-mmm-yyyy)	|__|__| - |__|__|__| - |2|0|1|__|	date_q
2	Child screened from which ward?	PSCW$_1$ A&E$_2$ Moyo$_3$	loc
3	Diagnosis at admission includes ARI?	No$_0$ Yes$_1$	scr_
4	Date of birth (dd-mmm-yyyy) (Child must be between 1 and 5 years old [inclusive] at the time of screening)	|___|___| - |___|___|___| - |___|___|___|___|	dob
5	If DOB unknown, age (years)		age
	Is child eligible?	No$_0$ Yes$_1$	cont

Preliminary information and child characteristics

#	Question	Response	Variable
6	Consent obtained? (If no voluntary written consent, end questionnaire)	No$_0$ Yes$_1$	cont
7	Participant ID (Attach label if available)		pid
8	SPINE number (Attach label if available)		spin
9	Gender	Male$_1$ Female$_2$	sex
	Diagnosis at QECH admission (at A&E) (One of these must be consistent with acute respiratory infection)		
10a	Diagnosis at admission-1		dxa_1
10b	Diagnosis at admission-2		dxa_2
10c	Diagnosis at admission-3		dxa_3
		Other$_5$ (specify):	dxa_other

PCV13 status

#	Question		Response	Variable
11	Did the child receive at least one dose of PCV13 vaccine?		No$_0$ Yes$_1$	pcv_vac
12	Date of dose 1 PCV13 (dd-mmm-yyyy)		|__|__| - |__|__|__| - |2|0|1|__|	dte_d1
13	Date of dose 2 PCV13 (dd-mmm-yyyy)	Not Received$_0$	|__|__| - |__|__|__| - |2|0|1|__|	dte_d2
14	Date of dose 3 PCV13 (dd-mmm-yyyy)	Not Received$_0$	|__|__| - |__|__|__| - |2|0|1|__|	dte_d3
15	PCV13 information confirmed by health passport or other document?		No$_0$ Yes$_1$	vacinfo
15a	Take a photo of the vaccination page (with PCV info.) of health passport		Not Done$_0$ Done$_1$	hpphoto

Medical History

#	Question	Response			Variable
16	Was child referred to QECH from health centre for this illness??	No$_0$	Yes$_1$		ref_q
16a	If yes, was the child provided any antibiotics at the health centre? **Note:** Specify using list of antibiotics in appendix 1; More than one answer is possible.	No$_0$	Yes$_1$ (Specify)		ab_hc
17	Did the child receive antibiotics from any other source (including a private pharmacy) within the 7 days prior to admission? **Note:** Specify using list of antibiotics in appendix 1; More than one answer is possible.	No$_0$	Yes$_1$ (Specify)		ab_other
18a	Antibiotics received after arriving at QECH **BUT** before NPS collected? (refer to patient file) **Note:** Specify using list of antibiotics in appendix 1; More than one answer is possible.	No$_0$	Yes$_1$ (Specify)		ab_pst
18b	If yes, please note the date and time of 1st dose of each antibiotic.	UNK$_{88}$	Date (yyyy-mm-dd)	Time (24-hr clock)	ab_tim
19	Is the child receiving TB treatment?	No$_0$	Yes$_1$	UNK$_{88}$	tb_tx
20a	Has child ever tested positive for HIV?	No$_0$	Yes$_1$	UNK$_{88}$	hivc
20b	If yes, date of positive HIV test? (dd-mmm-yyyy)	UNK$_{88}$	|__|__| - |__|__|__| - |2|0|1|__|		hdte_c
20c	Child's HIV test result documentation seen or verbal?	Seen$_1$	Verbal$_2$		chiv_hp
21a	Has mother ever tested positive for HIV?	No$_0$	Yes$_1$	UNK$_{88}$	hivm

21b	If yes, date of positive HIV test? (dd-mmm-yyyy)	UNK$_{88}$	|___|___| - |___|___|___| - |2|0|1|___|		mdte_d2
21c	If mother's HIV test date unknown, was it prior to child's (participant's) birth?	No$_0$	Yes$_1$	UNK$_{88}$	mhiv_hp
21d	Mother's HIV test result documentation seen or verbal?	Seen$_1$	Verbal$_2$		mhiv_hp

22a	Does the child have any other immunocompromising illness?	No$_0$	Yes$_1$	UNK$_{88}$	imm
22b	If yes, specify:				imm_sp

Vaccine status

		Vaccines received (Circle answer)			Date of Vaccination (dd-mmm-yyyy)	
23a	BCG	No$_0$ Yes$_1$ UNK$_{88}$	bcg	23b	|___|___| - |___|___|___| - |___|___|___|___|	bcgdat
24a	Polio-0	No$_0$ Yes$_1$ UNK$_{88}$	pol0	24b	|___|___| - |___|___|___| - |___|___|___|___|	pol0dat
25a	Polio-1	No$_0$ Yes$_1$ UNK$_{88}$	pol1	25b	|___|___| - |___|___|___| - |___|___|___|___|	pol1dat
26a	Polio-2	No$_0$ Yes$_1$ UNK$_{88}$	pol2	26b	|___|___| - |___|___|___| - |___|___|___|___|	pol2dat
27a	Polio-3	No$_0$ Yes$_1$ UNK$_{88}$	pol3	27b	|___|___| - |___|___|___| - |___|___|___|___|	pol3dat
28a	DPT-1	No$_0$ Yes$_1$ UNK$_{88}$	dpt1	28b	|___|___| - |___|___|___| - |___|___|___|___|	dpt1dat
29a	DPT-2	No$_0$ Yes$_1$ UNK$_{88}$	dpt2	29b	|___|___| - |___|___|___| - |___|___|___|___|	dpt2dat
30a	DPT-3	No$_0$ Yes$_1$ UNK$_{88}$	dpt3	30b	|___|___| - |___|___|___| - |___|___|___|___|	dpt3dat
31a	Measles	No$_0$ Yes$_1$ UNK$_{88}$	measl	31b	|___|___| - |___|___|___| - |___|___|___|___|	measldat
32a	RV-1	No$_0$ Yes$_1$ UNK$_{88}$	rv1	32b	|___|___| - |___|___|___| - |___|___|___|___|	rv1dat
33a	RV-2	No$_0$ Yes$_1$ UNK$_{88}$	rv2	33b	|___|___| - |___|___|___| - |___|___|___|___|	rv2dat

34	Vaccination status information (Q. #23 - #33) confirmed by HP or other document?	No$_0$	Yes$_1$	hypox

Clinical Presentation (Refer to patient file)

35a	Fever present?<br>**Note:** Yes, if reported on admission form or temperature ≥37.5°C documented since admission.	No$_0$	Yes$_1$	UNK$_{88}$	fvr
35b	If yes, how many days ago did fever start?	days			fvr_dur
36a	Cough present (persistent and duration <14 days)?<br>**Note:** Yes, if reported on admission form or documented since admission.	No$_0$	Yes$_1$	UNK$_{88}$	cgh
36b	If yes, how many days ago did cough start?	days			cgh_dur

Difficulty Breathing

37a	Fast breathing *(≥40 breaths/min*ute)?<br>**Note:** Yes, if reported on admission form or documented since admission.	No$_0$	Yes$_1$	UNK$_{88}$	fast_br
37b	If yes, breaths/min?<br>**Note:** Record highest respiratory rate since admission.	breaths/min			rr

38	Chest indrawing?<br>**Note:** Yes, if documented since admission.	No0	Yes1	UNK88	chst_in
39	Stridor (in a calm child)?<br>**Note:** Yes, if documented since admission.	No0	Yes1	UNK88	strdr
40	Apnoea?<br>**Note:** Yes, if documented since admission.	No0	Yes1		apn
41	Nasal flaring?<br>**Note:** Yes, if documented since admission.	No0	Yes1		nflr
42	Wheeze?<br>**Note:** Yes, if documented since admission.	No0	Yes1		wheez
43	Bronchial breathing?<br>**Note:** Yes, if documented since admission.	No0	Yes1		bbrth
44	Reduced air entry?<br>**Note:** Yes, if documented since admission.	No0	Yes1		redair
45	SpO_2 (%)<br>**Note:** Record lowest documented since admission.	%			sp
46	Hypoxia (O_2 saturation <92%)?<br>**Note:** Yes, if documented since admission.	No0	Yes1		hypox
47	Received oxygen?	No0	Yes1		o2

Household information

48	Does child live in an institution (e.g. orphanage)?	No0	Yes1	c_inst
49	Name of Blantyre suburb/township where child lives			town
50	GPS coordinates (using ePAL; no home visit)	**lat**|__|__|.|__|__|/ **lon**|__|__|.|__|__|		lat / long
51	How many bedrooms in your house (minimum 1)?	|___|___|		Rooms
52	Who lives at home?<br>Number of adults (16 years and older)	|___|___|		noadults
53	Number of children 5-15 years of age	|___|___|		nochild15
54	Number of children 0-4 years of age	|___|___|		nochild5
55	Amongst the children, how many born after 1 October 2011?			oct_11

56a	Child 1, received PCV dose-1	No0	Yes1	UNK88	d1_1
56b	Child 1, received PCV dose-2	No0	Yes1	UNK88	d2_1
56c	Child 1, received PCV dose-3	No0	Yes1	UNK88	d3_1
56d	HP or other document available to confirm PCV history?	No0	Yes1		hp_1
57a	Child 2, received PCV dose-1	No0	Yes1	UNK88	d1_2
57b	Child 2, received PCV dose-2	No0	Yes1	UNK88	d2_2
57c	Child 2, received PCV dose-3	No0	Yes1	UNK88	d3_2
57d	HP or other document present to confirm PCV history?	No0	Yes1		hp_2
58a	Child 3, received PCV dose-1	No0	Yes1	UNK88	d1_3
58b	Child 3, received PCV dose-2	No0	Yes1	UNK88	d2_3
58c	Child 3, received PCV dose-3	No0	Yes1	UNK88	d3_3
58d	HP or other document present to confirm PCV history?	No0	Yes1		hp_3
59a	Child 4, received PCV dose-1	No0	Yes1	UNK88	d1_4
59b	Child 4, received PCV dose-2	No0	Yes1	UNK88	d2_4
59c	Child 4, received PCV dose-3	No0	Yes1	UNK88	d3_4
59d	HP or other document present to confirm PCV history?	No0	Yes1		hp_4

Antibiotic exposure

60	Chest X-ray completed?	No0	Yes1	UNK88	X_comp

60a	If yes, digital X-ray or plain film only?	Digital$_0$	Plain$_1$	X_type
60b	If no X-ray, specify why not:			X_reas
61	NP swab taken?	No$_0$	Yes$_1$	nps
61a	If no NP swab was collected, specify why not:			npsreas
61b	Was NP swab collection adequate?<br>Hint: Did you feel resistance and was swab at back of nasopharynx for 3-5 seconds?	No$_0$	Yes$_1$	npsad
61c	Is there blood on the swab?	No$_0$	Yes$_1$	npsbld
61d	Is there nasal mucus on the swab?	No$_0$	Yes$_1$	npsmuc
62a	Urine sample collected?	No$_0$	Yes$_1$	urine
62b	If yes, please note the date and time that urine sample was passed.	Date (yyyy-mm-dd)	Time (24-hr clock)	ur_tim
63	Form completed by: code	|___|___|___|		Enum
	Form completed by: signature			

Appendix 1: Antibiotics listing for Questions 16a, 17 and 18a

1. Amoxicillin
2. Benzylpenicillin
3. Ceftriaxone
4. Chloramphenicol
5. Ciprofloxacin
6. Cotrimoxazole (Bactrim)
7. Erythromycin
8. Gentamicin
9. Metronidazole (Flagyl)
10. Other antibiotic, specify: _______
