# Supplementary material for: Urine bioassay optimisation to assess the association between antimicrobial exposure, pneumococcal carriage and antimicrobial resistance among hospitalised children in Malawi
Source: BMC Infect Dis. 2025 Oct 13;25:1291. doi: 10.1186/s12879-025-11871-w (PMC12516879; doi:10.1186/s12879-025-11871-w)
Supplement: Supplementary file 2 — Supplementary Material 2. [file 12879_2025_11871_MOESM2_ESM.pdf]

PCVPA Study
Malawi-Liverpool-Wellcome Trust Clinical Research Programme
P.O. Box 30096, Chichiri, Blantyre 3, Malawi

SOP: Urine sample collection among children <5 years at QECH

At recruitment:

- After consent, give a urine collection container to the parent/guardian.
- Provide the parent/guardian with a pen and ask her/him to note the time when the urine is passed onto the urine collection tube.
- On Recruitment Log form, record (Yes or No) whether the child was given antibiotics at the A&E, or at the PSCW.
- If yes, record date and time the child was given the 1st dose of antibiotics, and the kind of antibiotic/s given.
- If the child did not receive antibiotics prior to recruitment (e.g., at A&E), encourage parent/guardian to collect a urine sample as soon as possible.

When collecting the urine sample:

- Record the date and time in the Recruitment Log when the urine was **passed**.
- If not done already, record the date and time when the **1st dose** of antibiotic was given, and record the antibiotic/s.
- Record all antibiotics given to the child **at QECH before** the urine was passed.

At discharge:

- Check the Patient File if any antibiotic treatment was given during the stay at QECH.
- On Recruitment Log form, record (Yes/No) whether the child was given antibiotics while at QECH.
- Record all antibiotics that were given during the hospital stay in the space provided on the form.

SOP: Standard Operating Procedure; A&E: Accident and Emergency Department; PSCW: Paediatric Special Care Ward; QECH: Queen Elizabeth Central Hospital
